# Natural Ventilation Characterization in a Classroom under Different Scenarios

**DOI:** 10.3390/ijerph18105425

**Published:** 2021-05-19

**Authors:** Sergio A. Chillon, Mikel Millan, Iñigo Aramendia, Unai Fernandez-Gamiz, Ekaitz Zulueta, Xabier Mendaza-Sagastizabal

**Affiliations:** 1 Nuclear Engineering and Fluid Mechanics Department, University of the Basque Country, UPV/EHU, Nieves Cano 12, Vitoria-Gasteiz, 01006 Araba, Spain; sachillon001@ikasle.ehu.eus (S.A.C.); inigo.aramendia@ehu.eus (I.A.); mendazaxabier@gmail.com (X.M.-S.); 2 System Engineering and Automation Control Department, University of the Basque Country, UPV/EHU, Nieves Cano 12, Vitoria-Gasteiz, 01006 Araba, Spain; mmillan011@ikasle.ehu.eus (M.M.); ekaitz.zulueta@ehu.eus (E.Z.)

**Keywords:** particle size, carbon dioxide concentration, CO_2_, classroom air composition, natural ventilation, Arduino, SCD30, APS, school health, COVID-19

## Abstract

The COVID-19 pandemic has pointed to the need to increase our knowledge in fields related to human breathing. In the present study, temperature, relative humidity, carbon dioxide (CO_2_) concentration, and median particle size diameter measurements were taken into account. These parameters were analyzed in a computer classroom with 15 subjects during a normal 90-minute class; all the subjects wore surgical masks. For measurements, Arduino YUN, Arduino UNO, and APS-3321 devices were used. Natural ventilation efficiency was checked in two different ventilation scenarios: only windows open and windows and doors open. The results show how ventilation affects the temperature, CO_2_ concentration, and median particle diameter size parameters. By contrast, the relative humidity depends more on the outdoor meteorological conditions. Both ventilation scenarios tend to create the same room conditions in terms of temperature, humidity, CO_2_ concentration, and particle size. Additionally, the evolution of CO_2_ concentration as well as the particle size distribution along the time was studied. Finally, the particulate matter (PM_2.5_) was investigated together with particle concentration. Both parameters showed a similar trend during the time of the experiments.

## 1. Introduction

The COVID-19 pandemic has greatly impacted both the global economy and the health of individuals [1]. As a result, it is essential to thoroughly examine possible methods of contagion in different situations. The most effective measures to prevent SARS-CoV-2 transmission are those that reduce interaction between humans such as social distancing, quarantine strategies, and transport restrictions [2]. Although Oliver et al. [3] mention that various vaccines have been developed, according to Engelbrecht et al. [4] and Renardy et al. [5], their availability for the whole population could fall behind as new waves of the virus emerge all around the world.

Consequently, Wang et al. [6] concluded that isolation measures are unsustainable in the long term because they slow economic growth and limit academic, industrial, and commercial activities. Therefore, other measures need to be implemented that allow for interaction in indoor atmospheres under restrictive scenarios, i.e., ventilation and CO_2_ measurement. For instance, Schiobula et al. [7] explained how monitoring CO_2_ and ventilation could be used to reduce the risk of COVID-19 contagion in indoor school environments.

According to Alcamí et al. [8], SARS-CoV-2 transmission methods include respiratory droplets, airborne transmission, direct contact between an infected person and another person, and indirect contact or fomites (which means that an infected person has touched a surface or has deposited droplets there). However, Zhang et al. [9] demonstrated that transmission through aerosols or airborne transmission is the main source.

Regarding the transmission risk, indoor environments pose a major SARS-CoV-2 infection risk. In fact, Qian et al. [10] concluded that most outbreaks in China in February 2020 happened in closed environments such as homes, public transport vehicles, and shopping or entertainment venues. 

According to Bartyzel et al. [11], a good ventilation system should be implemented to avoid high transmission in an indoor environment. This is even more crucial in environments where many people need to coexist for several hours. Poor air quality is usually understood as an excessively high concentration of particulate matter (PM). However, CO_2_ is also a critical component with respect to the quality of indoor air and should be reduced via ventilation. This phenomenon is intensified in classrooms, which are usually crowded, overheated, and poorly ventilated. This results in a possible increase in carbon dioxide (CO_2_), which could be dangerous if the concentration level exceeds the value of 0.15 percentage value of CO_2_ (1500 ppm) or even lower levels (1000 ppm). According to Persily [12], indoor carbon dioxide concentrations are important and have played a role in discussions related to ventilation and indoor air quality (IAQ). The relevance of CO_2_ concentrations to ventilation and IAQ standards is based on their correlation to indoor levels of bio-effluents and associated odors and to ventilation rates per person. Hou et al. [13] summarized the main studies focusing on the indoor air quality of classrooms. They also studied the correlation of CO_2_ and PM_2.5_ (indoor and outdoor ratios) concentrations in classrooms, relating the data to the ventilation rates in classrooms and the classroom occupancy. Rumchev et al. [14] also concluded that populations with a high rate of poverty are a greater risk of suffering problems related to lung health due to the lack of basic ventilation equipment such as chimneys and even windows.

In order to maintain desirable air quality in indoor classrooms, Morawska et al. [15] recommend taking very simple measures such as avoiding overcrowding, ensuring high ventilation rates and installing supplementary ventilation systems with air cleaners. In the study of Pulimeno et al. [16], more comprehensive measures were also proposed to achieve optimal IAQ, such as keeping equipment that produces particles and VOCs outside the classroom, monitoring the classroom temperature with thermostats, and, whenever possible, performing annual monitoring of the indoor PM and radon concentrations. Morawska et al. [15] also recommended installing high performance filter systems for air conditioners and air purifiers.

With the purpose of measuring the air quality and the efficiency of ventilation, Dinh et al. [17] noted that CO_2_ should be measured as an indicator. If ventilation is controlled by the monitoring of CO_2_ levels in indoor environments, people can be prevented from acquiring many diseases, especially those related to respiratory viral infections. CO_2_ is an important parameter to measure because it is a marker of the air that has been exhaled and can be linked to the probability of infection through the Well‒Riley equation. CO_2_ particles are denser than air; however, they are carried with the flow as virus particles would be. CO_2_ being higher than a certain level indicates that the ventilation is inadequate, and measures should be taken. Bhagat et al. [18] determined that one of the most efficient strategies for reducing exposure risk seems to be displacement ventilation. When this strategy is designed properly, it helps with vertical stratification and removes polluted warm air near the ceiling. Additionally, wearing face coverings is an effective measure as it reduces the direct ejection of droplets and bio-aerosols. Furthermore, they recommended avoiding mixed ventilation strategies, since that can distribute air through a room and does not provide clean zones. Wang et al. [19] discussed the feasibility of displacement ventilation in schools along with a heat recovery pump. Through this system, classroom air quality has been enhanced, while the energy consumption of the building is reduced. Computational fluid dynamics software (CFD) was used to demonstrate the technical feasibility of this development. Li et al. [20] introduced an innovative alternative ventilator, replacing one part of the window frame with a ventilator whose core component is a cross-flow fan (CFF).

However, Dominguez-Amarillo et al. [21] agreed that there are many issues that impede adequate ventilation. Many buildings have low radiant temperatures due to the limited performance of thermal building envelopes. Therefore, users limit the voluntary ventilation times; this can even worsen in SARS-CoV-2 confinement scenarios during colder periods. Satish et al. [22] realized that, at other times, the aim is just reducing energy consumption, which leads to low rates of ventilation. 

In recent years, new numerical techniques have been developed and found to be useful in investigating physical parameters that involve public health. The most used numerical techniques include CFD technology; see Chillon et al. [23]. Several researchers revealed important information about the exhalation of microparticles during different breathing actions. In this manner, Dbouk et al. [24] demonstrated that 2 m is a safe face-to-face distance between humans to prevent COVID-19 infections only in no stream situations. The same authors corroborated in [25] that using face masks greatly reduces the number of released droplets in the environment. Using face masks also increases droplets’ evaporation ratio, due to the fact that droplet clouds are less dense. According to Feng et al. [26], relative humidity (RH) does not affect the droplets’ trajectories but interferes with their evaporation. Aliabadi et al. [27] mentioned that small droplets evaporate more quickly at low RH. Vuorinen et al. [28] added that complete droplet evaporation leads to the appearance of a solid residue. These residues accumulate, forming a suspended cloud called an aerosol. These aerosols travel on air streams. Yang et al. [29] recommended controlling indoor RH to control this manner of spreading droplets. On the other hand, Shao et al. [30] suggested designing effective ventilation strategies to disperse aerosol clouds safely.

The aim of the present work is to investigate CO_2_ concentration in addition to ambient temperature, relative humidity, particles size, PM_2.5_ and particle concentrations in a classroom under different ventilation scenarios and conditions. The acquired data contributes as a guideline to define ventilation strategies and to improve IAQ in academic installations. The first scenario has 15 people inside the classroom, all of them wearing surgical masks the whole time. The second studies the same parameters with the room empty. The same ventilation conditions were reproduced during both experiments for 90 min, separated into four time stretches. During the first and the third time stretches no ventilation existed. During second time lapse, windows were opened. For the last time stretch, ventilation was increased by opening windows and doors.

## 2. Materials and Methods

The experiments were all conducted in computer room 1.3 of the Faculty of Engineering at the University of the Basque Country UPV/EHU of Vitoria-Gasteiz. The classroom area is 91.8 m^2^. Data were collected on two different days in November 2020. The first collection was on Friday 27th in the morning, and the second on Monday 30th in the afternoon. The morning experiment was carried out with 15 people inside (14 students and the teacher). The age of the subjects was 19 to 23 for the student subgroup, and the teacher was 44 years old. The participants were 10 males and five females, arbitrarily distributed, but complying with the order of May 19th SND/422/2020 relative to the COVID-19 pandemic. According to this order, participants wore surgical or clinical masks the whole time, and the distances between them were as large as possible: around 1.5 m between students who shared a desk and around 2 m between students who were sitting at different desks, laterally measured. During the experiment, students attended a practical computer class lesson and were able to interact with each other but without leaving their workstations. At the end of the event, desks, keyboards, mice, and screens were disinfected in the same manner as they were at the beginning. The afternoon experiment was conducted with the classroom empty.

In both cases, the ventilation was natural: opening doors and/or windows. The outdoor conditions were similar in both cases, with the only difference being the wind speed and direction. During the experiment with the populated classroom, the average wind velocity was 12.4 m/s in a southeasterly direction. At the time of the second experiment, when the classroom was empty, the wind speed was 6 m/s in the northwest direction. Windows were facing west. Data were collected by the Basque meteorology service, EUSKALMET, from the nearest weather station. For more details, see Table 1.

CO_2_ concentration, temperature, and relative humidity measurements were carried out with two SCD30 sensors. These modules can measure the temperature, humidity, and CO_2_ concentration in the air using NDIR technology. The CO_2_ sensor ranges from 400 ppm to 10,000 ppm, and has an error of ±(30 ppm + %3 MV). The temperature sensor ranges from −40 °C to 70 °C. Between 0 °C and 50 °C, the sensor has an error of ±(0.4 °C + 0.023 × (T[°C] − 25 °C)). The humidity sensor ranges from 0% to 100% and, at 25 °C, has an error of ±3% RH. The sensors take measurements at intervals of 2 s. The SCD30 sensors (Sensirion AG, Stäfa, Switzerland) were calibrated using the SparkFun SCD30 library with the default parameters and following the instructions found in the sensors’ datasheet. The automatic self-calibration (ASC) routine was performed for the recommended time period of seven days, with at least 1 h a day of fresh air. Both sensors remained connected to the power supply for the whole duration of the ASC. 

**Figure 1:**
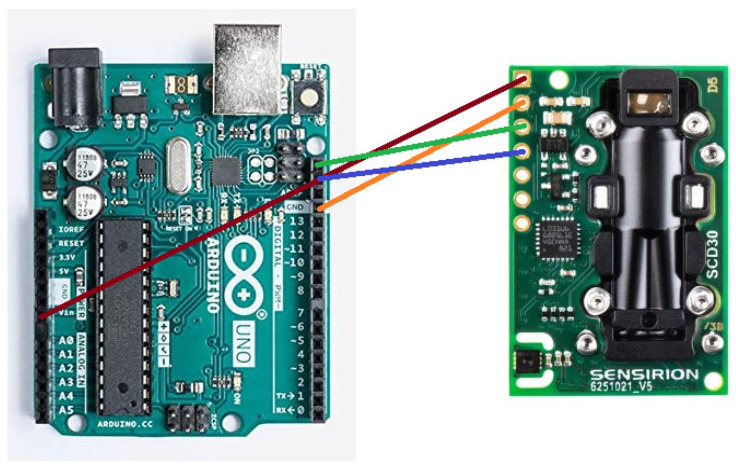
The connection between Arduino Uno and SCD30. Red cable is Vin, orange is GND, green is SCL, and blue is SDA. For YUN, the same configuration was used but two 1 KΩ pull-up resistors were needed.

Each SCD30 sensor was connected to an Arduino (YUN REV2 or UNO R3). Data were transferred from the sensor to the Arduino following the I2C protocol (see Figure 1). As previously stated, the SCD30 sensor sent data every 2 s; however, measurements were taken every 6 s. The Arduinos were programmed with the SparkFun library, specially conceived for the SCD30 sensor.

Each Arduino was connected via USB to a computer. Data were transferred from the Arduino to the computer every 6 s via the serial port. The transferred data were then processed on the computer using the Matlab environment. Furthermore, a GUI was developed on Matlab that graphically displays the temperature, humidity, and CO_2_ in real time for each sensor. Once the experiment was concluded, the data were saved in an Excel file.

**Figure 2:**
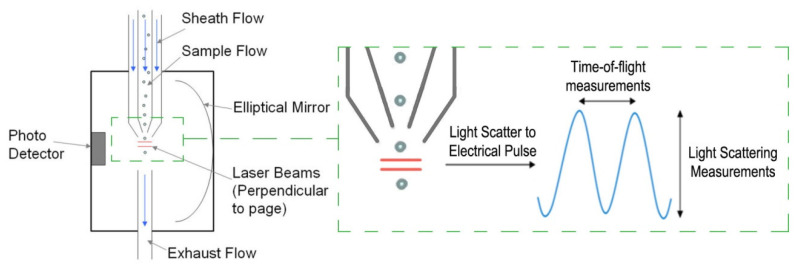
Aerodynamic Particle Sizer (APS) operation scheme and a detailed view with aerosol droplets crossing the overlapping breams and generating the double-crested signal.

Between different techniques of particle sizing, the aerodynamic approach was chosen for the current study. Therefore, an Aerodynamic Particle Sizer (APS) spectrometer was used to measure the aerosol microparticles’ sizes inside the classroom. This device model (APS-3321; TSI Incorporated, MN, USA) was successfully used in Aramendia et al. [31] for measuring the nebulized particle size distributions. The aerosol, drawn into the APS inlet, is immediately split into a sample flow through the inner nozzle, and a sheath flow through the outer nozzle. This device generates a signal every time a particle crosses two laser beams placed within the inlet nozzle, providing high-resolution measurements for droplets between 0.5 and 20 µm (see Figure 2). The acceleration of droplets, due to inertia, is smaller for larger droplets. Therefore, the APS theory operation to calculate this acceleration is based on the time between the peaks of the signal produced by the two laser beams, also known as the time of flight. Then, the APS memory, which must initially be calibrated, converts each time-of-flight measurement recorded to the corresponding aerodynamic particle diameter, described as the diameter of a spherical particle with a density of a water droplet (1000 kg/m^3^) that has the same settling velocity as the measured particle. Pfeifer et al. [32] noticed that the sizing error rarely exceeds 10%.

Collected data were acquired, analyzed, and stored by means of the APS 3321 processing software package AIM (TSI Incorporated). Chen et al. [33] described the mathematical models that AIM software used to calculate the quantity of suctioned particles and their size. APS converts each time-of-flight measurement into its corresponding aerodynamic particle diameter (*D_a_*). The relationship between this parameter and the geometric particle diameter (*D_g_*) is given by Equation (1):

(1) $$D_{g}=D_{a}\;\sqrt{\frac{\rho_{0}}{\rho }}$$

where *ρ* is the density of the measured particles and *ρ*_0_ is the unit density (1 g/cm^3^).

The primary measurement of the APS 3321 is the concentration (*N*), expressed as particles/cm^3^, where the total number of particles per unit volume of air sampled is measured for a given channel; see Equation (2):

(2) $$N=\overset{u}{\underset{l}{\sum }}n$$

where *u* and *l* are the upper and lower channel boundary, respectively, and *n* is the number-weighted concentration per channel.

The Mass Median Aerodynamic Diameter (MMAD), which measures the aerodynamic diameter that divides the aerosol size distribution in half, has also been obtained to evaluate the concentration of particulate matter (PM). In particular, we assessed PM_2.5_, known as fine particles, i.e., those that have diameter values less than 2.5 µm and are related with health issues.

The duration of the experiments depended on the duration of the ongoing lecture in the classroom, which mostly ranged from 1 h to 2.5 h. Moreover, the program was designed in a flexible way. Indeed, the GUI allowed the user, via a toggle button, to start and end the experiment as needed.

**Figure 3:**
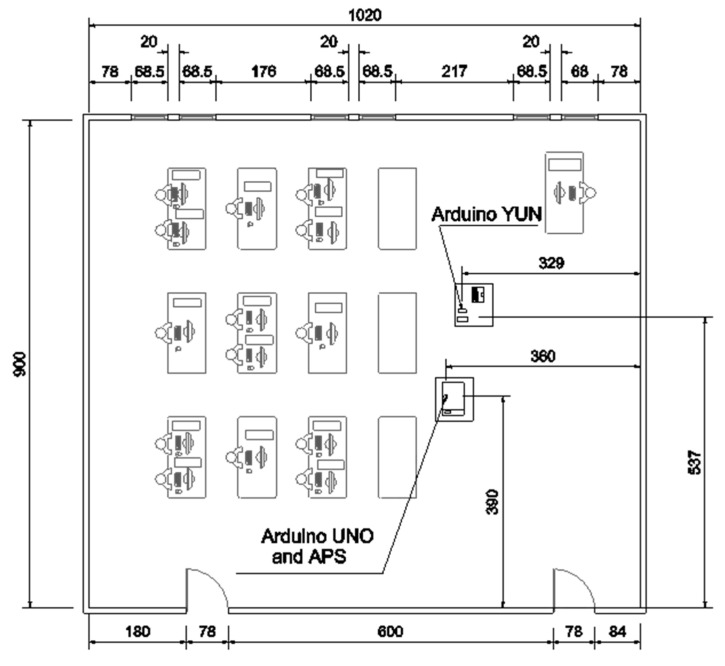
Layout of the classroom. All measurements are in cm. The room area is 91.8 m^2^.

Figure 3 illustrates the layout of the Info 1.3 room. The dimensions of the room are 900 cm × 1020 cm × 264 cm; it contains six 68.5 cm × 117.5 cm windows and two 78 cm × 200 cm doors. 

## 3. Results

The experiment consisted of two sets of measurements in the same room, shown in Figure 3. The first set was performed while the room had 15 persons inside, and the second set was performed while the room was empty. Both sets of measurements were completed using the same procedure: for the first 13 min, the room’s windows and doors were closed. For the following 27 min, the windows were open, while the doors remained closed. For the next 33 min, the windows and doors were shut. Finally, the windows and doors were open for the remainder of the experiment. Each set of measurements lasted 1 h 30 min and data were collected with two SCD30 sensors and an APS. The two SCD30 took a sample every 6 s, while the APS took a sample every 2 min.

### 3.1. Temperature and Humidity

**Figure 4:**
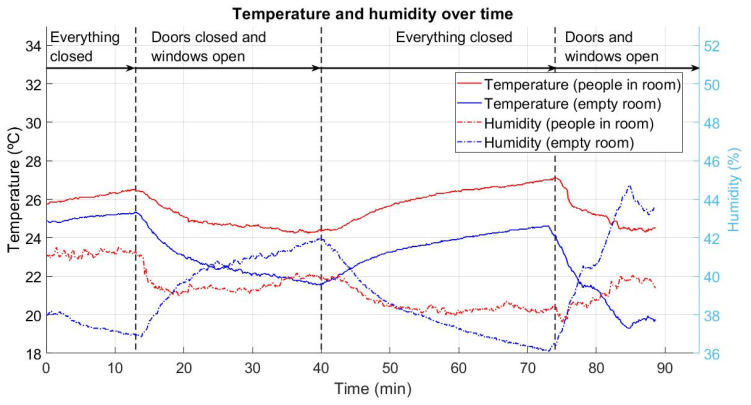
Temperature (°C, continuous line) and relative humidity (%, discontinuous line). Red lines correspond to the populated room (15 people) scenario. Blue lines correspond to the empty room scenario. First time stretch: closed room; second time stretch: windows open; third time stretch: closed room; fourth closed room: windows and doors open.

Figure 4 illustrates the temperature (T) measurements on the left side and relative humidity (RH) on the right side, in the four different scenarios mentioned above (doors and windows closed, only windows open, doors and windows closed, and doors and windows open). This graph shows measurements taken during both experiments (15 people inside the room versus empty room). Both T and RH data were obtained via a SCD30 device. 

In the case of people inside, the first 13 min represents the time from the entrance of the 15 subjects into the room, previously ventilated and with the doors and windows closed, until the opening of windows at minute 13. During this period, the temperature sensor showed an increasing line, with increments of 1 °C, going up to T = 26.5 °C. A relative humidity meter showed irregular lines full of peaks and valleys around RH = 41.1%. In the empty room, T increased gently, in 0.5 °C increments, to T = 24.8 °C. On the other hand, RH showed a decreasing trend that was more continuous and less variable than during the scenario with people inside, decreasing by increments of 1.2% to RH = 36.8%.

In minute 13 windows were opened getting a ventilation area of 4.83 m^2^. In the case of people inside, temperature immediately decreased progressively for 2.2 °C. RH also experimented a sudden decrease of 2%, to RH = 39%, and afterwards it started to oscillate softly gently until recover RH = 40% in minute 40. In terms of empty room scenario, temperature showed a similar trending as in people inside scenario. Temperature decreased 3.7 °C grades. As far as humidity is concerned, in contrast with the case with people in room, it strongly increased for almost 3% in only seven minutes after windows were opened. In next 20 min, until minute 40, relative humidity continued increasing 2% more.

In the third time stretch, the windows were closed again from minute 40 to minute 73. For the case with people in the room, the temperature increased by 3.8 °C during the 33 min. The humidity decreased from RH = 40% to RH = 38.1%. In the empty room scenario, the temperature increased by 3 °C in the same pattern as in the scenario with people in the room, with the same temperature difference at each moment. In contrast, the humidity suffered an abrupt decrease of almost 5%.

In the last ventilation experiment time stretch, from minute 73 to minute 90, the doors and windows were open. For the people inside scenario, the temperature experienced a more abrupt decrease of 2.5 °C, reaching 24.5 °C. This seemed to be the natural temperature of the room while it was being ventilated with people inside. The relative humidity decreased in 1 min from RH = 38.1% to RH = 37.6%, and recovered during the next 1 min, reaching RH = 39.9%. In the empty room case, the temperature dropped 5 °C in 12 min. In contrast, the relative humidity rose rapidly, increasing by 8% in 12 min.

### 3.2. CO_2_ Concentration and Median Particle Diameter

**Figure 5:**
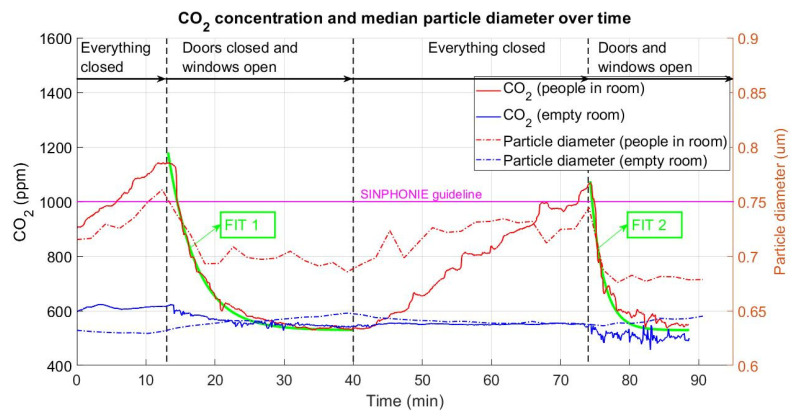
CO_2_ concentration (ppm, continuous line) and particle median diameter (µm, discontinuous line). Red lines correspond to a populated room scenario (15 people). Blue lines correspond to the empty room scenario. First time stretch: closed room; second time stretch: windows open; third time stretch: closed room; fourth time stretch: windows and doors open.

Figure 5 shows, on the left side, the evolution of the CO_2_ concentration in parts per million (ppm) and the median size of particles (in µm) on the right side. CO_2_ measurements were taken using a SCD30 device and particle size measurements were taken via APS-3321. During the first 13 min, from the entry of the 15 subjects into the room in a no-ventilation situation until the opening of windows, a quick and linear increase of CO_2_ was observed, from 904 ppm until 1140 ppm. Additionally, an increase in the median particle size was observed, from 0.71 µm to 0.76 µm. Both measures, CO_2_ concentration and median particle size, were absolute maximum measures in the recorded series. In the empty room scenario, as expected, the measured concentrations of CO_2_ were a quasicontinuous value that varied between 597 and 624 ppm. These small variations could be generated by the people who set up the APS device in the first instance and by the entrance of the person who opened the windows at minute 13. As for particle diameters, a continuous trend was observed, with a usual value of 0.63 µm.

During the next 27 min, until minute 40, the windows were completely open. In the case where people were in the room, 2 min after the windows were opened, a marked decrease in the CO_2_ concentration was observed. This decrease was exponential from 1140 ppm until it reached a horizontal asymptote near 535 ppm from minute 30 onwards. In terms of the particle diameter, the median value decreased from the mentioned 0.76 µm at minute 13 to 0.69 µm at minute 18, with some small valleys and peaks appearing thereafter. In the case of an empty room, a gradual CO_2_ decrease was detected. The concentration gradually decreased from 622 ppm to 543 ppm. This decrease showed a large number of small peaks and valleys, which were interpreted as interior–exterior gas exchange. Note that the effect of opening the windows on the linear increase in the median particle diameter was remarkable: it went from 0.63 µm to 0.65 µm.

At minute 40, the windows were closed again, cutting off the room from airflow for 33 min, until minute 73. During this time, a linear increase with some irregularities was shown. The CO_2_ concentration increased from 535 ppm to 1070 ppm. With respect to the median particle diameter value, it increased irregularly from the minimum (0.68 µm) at minute 40 to 0.74 µm at minute 73. There was only existed a brief period that could be considered stable during the three measurements, at minutes 52 to 64 with a value of 0.73 µm. In the empty room experiment, the CO_2_ concentration was completely stable, at 548 ppm. The median particle size decreased slowly and continuously from 0.65 µm to 0.63 µm.

During the last 17 min, minutes 73 to 90, both doors and windows were open. In the experiment with people in the room, a steep decrease in the CO_2_ concentration, with small peaks, was seen. In just 3 min, the concentration decreased from 1070 ppm to 601 ppm. This value continued decreasing but in a less pronounced way, until the asymptotic minimum of 546 ppm. It is important to note that this value was reached in both ventilation scenarios. In terms of the median particle diameter, a pronounced reduction in size was observed, from 0.74 µm to an exponential decrease of around 0.68 µm. For the empty room case, the trends in the windows-open scenario were repeated, i.e., a gradual decrease in the CO_2_ concentration, with pronounced oscillations between 550 ppm and 458 ppm. When doors and windows were open, the oscillations were much more pronounced and irregular. The particle median diameter value showed a small increase from 0.63 to almost 0.64 µm, reaching the value at the end of the second time stretch, with only windows open.

A guideline was marked in the maximum recommended value. According to Schools Indoor Pollution & Health Observatory Network in Europe (SINPHONIE) [34], this value should not cross the value of 1000 ppm. During the carried experiment, this value was exceeded twice, both in the everything closed scenarios.

An exponential fitting was performed on CO_2_ measures in the scenario with people in the room while the doors were closed and the windows open, or the doors and windows open (minute 13 to minute 40, and minute 73 to minute 90). Fittings are described by Equation (3):

(3) $$f\left(x\right)=a\cdot e^{bx},$$

where a and b are the coefficients of the fitting equation and f(x) corresponds to the CO_2_ concentration over time, represented by x. Table 2 presents the main parameters of both fittings.

Pandemic-related problems have created a great necessity of knowledge increasing about COVID-19 spreading. Dbouk et al. [24] studied the relationship between RH and particle sizes supposing that larger droplets are denser in virus population. Yang et al. [35] demonstrated that larger droplets have more axial penetration. On the other hand, according to van Doremalen et al. [35] viruses remain for hours in expelled aerosols. Lelieveld et al. [36] mentioned that 80–90% of exhausted particles during breathing actions are considerable as aerosol. Zheng et al. [37] declared CO_2_ concentration values as occupancy measure, and Lazović et al. [38] found a relationship between CO_2_ concentration and suspended micro particles. According to the study carried out by Setti et al. [39] in the north of Italy, the relation between high levels of CO_2_ and virus spreading was not obvious. With this background and obtained results in the current study, it seems interesting to deepen in the possible CO_2_ concentration and contagiousness with the aim to identify potentially risk scenarios so indoor as outdoor.

### 3.3. Particulate Matter 2.5 and Particle Concentration

**Figure 6:**
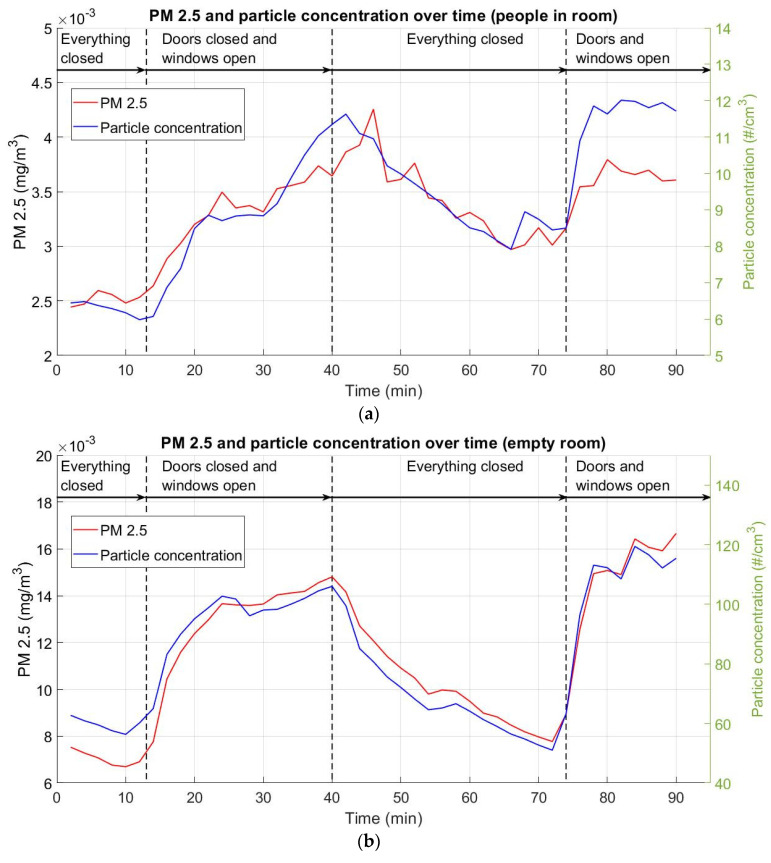
PM_2.5_ concentration (mg/m^3^, red line) and particle concentration (amount per cm^3^, blue line). First time stretch: closed room; second time stretch: windows open; third time stretch: closed room; fourth time stretch: windows and doors open. (**a**) People in room; (**b**) empty room.

Figure 6 represents the evolution of particulate matter 2.5 (PM_2.5_) measured in mg/m^3^ on the left axis and particle concentration in number of particles (cm^3^) on the right axis. In both cases, the observed trends were similar. This was an increase in concentrations of PM_2.5_ and particles in ventilation situations and decreases in situations with a lack of ventilation. At higher ventilation rates, with windows and doors open, the increasing gradients were higher than at slower ventilation rates or in no-ventilation situations. Figure 6 illustrates how, in the scenario with people inside, the PM_2.5_ and particle concentration values showed some irregularities between them. In contrast, in the empty classroom scenario, similar trends in PM_2.5_ and particle concentration values over time were seen in the four proposed ventilation situations. Note that, in outdoor ventilation situations, PM_2.5_ values tend to be the same as outdoor measured values, as assessed by the Basque meteorology service, EUSKALMET. According to the nearest meteorological station, PM_2.5_ values were 6·10^−3^ mg/m^3^ when people-inside experiment (November 27) and 14·10^−3^ mg/m^3^ when the experiment was carried out in an empty classroom (November 30).

## 4. Conclusions

In the current study, temperature, relative humidity (RH), CO_2_ concentration, and median particle diameter size relationships were investigated in a classroom of 91.8 m^2^. The measurements were carried out in two different scenarios: with 15 people inside the classroom and with an empty classroom. Each experiment lasted 90 min. The 1.5 h was divided into four time stretches: for the first 13 min, the classroom had no ventilation. For the next 27 min, natural, continuous ventilation was maintained by opening the windows. During the next 33 min, the room was again without ventilation. For the last 13 min, doors and windows were completely open, increasing the natural ventilation ratio to the maximum.

The results lead us to draw several conclusions:

- The temperature showed the same behavior in both cases (people in room and empty room). In the empty room case, the temperature was 1.5–3 °C lower. Natural ventilation cooled the room by 2–4 °C, depending on the occupancy and ventilation rate.
- The relative humidity results revealed a tendency towards equilibrium. The equilibrium point varied according to the ventilation conditions. After opening the windows, the RH readjusted to reflect the external conditions. This reconditioning is more significant at higher ventilation rates.
- In terms of CO_2_ concentration, a linear time increase was observed in the no-ventilation situation with people inside. This can be explained by the fact that the main CO_2_ source was the subjects. In both ventilation situations, an asymptotic decrease 570 ppm of CO_2_. Note that this value is close to the continuous one in the empty room scenario. In 15 min of ventilation with windows open, the CO_2_ minimum value was reached, versus within 5 min with both doors and windows open.
- The particles’ median diameter size showed a similar trend to the CO_2_ concentration. The median particle diameter size increased during no-ventilation situations and decreased significantly during ventilation states, especially at higher ventilation ratios.
- Finally, particulate matter PM_2.5_ and particle concentration were studied. Both parameters follow a similar trend during the time of experiment. Initially, with a no-ventilation, a decrease in both PM_2.5_ and particle concentration were observed. On the other hand, when natural ventilation was applied, a noticeable increase was found.

## Figures and Tables

**Table 1:** Experimental parameters and weather conditions.

No.	Measurement Day	Measurement Period	Classroom	Ventilation System	Classroom Occupancy	Temperature Outside	Relative Humidity Outside
1	27 November 2020	9:29–10:59	Info 1.3	natural	15	6 °C	79%
2	30 November 2020	17:04–18:34	Info 1.3	natural	0	7 °C	100%

**Table 2:** Fitting parameters and confidence values.

FIT	a	b	Confidence Bound	$R^{2}$	RMSE
FIT 1	24.83	−1.897	95%	0.9897	15.33
FIT 2	11.13	−2.271	95%	0.9595	24.34

## Data Availability

The data presented in this study are available on request from the corresponding author.
